# Personalized Medicine in Otolaryngology: Special Topic Otology

**DOI:** 10.3390/jpm12111820

**Published:** 2022-11-02

**Authors:** Georg Mathias Sprinzl, Astrid Magele

**Affiliations:** 1 University Clinic St. Poelten, Department of Otorhinolaryngology, Head & Neck Surgery, Dunant-Platz 1, 3100 St. Pölten, Austria; 2 Karl Landsteiner Institute of Implantable Hearing Devices, Henri Dunant Platz 1, 3100 St. Pölten, Austria

Globally, more than 1.5 billion people experience some degree of hearing loss. Of these, an estimated 432 million adults and 34 million children suffer from disabling hearing loss, that is, hearing loss of moderate or higher severity in the better hearing ear [1]. Disabling hearing loss and no benefit from conventional hearing aids are essential indications for implantable hearing systems, and the hearing threshold, the underlying pathology, and the anatomical conditions of the patients dictate the type of hearing implant indicated. 

Personalized medicine (PM) refers to the tailoring of medical treatment to the individual characteristics of each patient. In the last approx. 10 years, PM has shifted the traditional “one size/drug fits all” approach into a more stratified therapeutic strategy which includes prevention, diagnostics, therapy/treatment, and rehabilitation (Figure 1). In the field of otology, PM has nowadays evolved to a tailormade standard of care to optimize treatment and ensure the safe and reliable application of hearing interventions. The major goal of all otologic procedures nowadays, whether surgical or conservative, is not just to restore hearing but to regain quality of life. These goals affect not only the victims, but also their family and friends, and even their work colleagues.

This editorial will delineate the approach of PM in the field of otology. Based on the development of better radiological diagnostics and the audiological assessment of hearing disorders in recent years, the approach of PM has become an integral part of modern otological treatment of patients. The evolution of data management in our field gives us information for more precise interventions to treat hearing disorders. 

Optimized diagnostics such as e-BERA and OAEs has modified the approach to treat patients with hearing disorders significantly. Due to more precise audiological diagnosis, we are able to treat patients sooner in their lives and, hence, elevate the outcomes in hearing rehabilitation.

The development of modern radiologic diagnostics with CT and MRI and digital volume tomography has enhanced the quality of surgical treatment approaches in many ways.

Over the years, all these developments have enabled hearing implant companies to tailor the product to the needs of the treated subjects [2,3,4,5,6]. The birth of cochlear implantation more than 40 years ago, with the concept of “one implant for all”, is a good example of how the situation has changed in the field of otology [7,8]. Nowadays, otologists have a great variety of sophisticated implantable solutions to choose from: the latest research is focused on preserving delicate natural cochlear structures, along with atraumatic electrode placement aiming to preserve residual hearing. Nowadays, patients with functional low-frequency hearing can utilize combined electric–acoustic stimulation (EAS) [9,10]. Research has shown many benefits, especially for music appreciation and hearing in challenging environments [11]. Additionally, with the application of presurgical planning tools targeting the appropriate angular insertion depth and allowing individualized selection of electrode arrays, this enables the maximization of cochlear coverage ensuring better and more natural sound quality [12,13]. The ever-expanding candidacy indications nowadays also allow for the treatment of candidates with a missing or very small hearing nerve or severely abnormal inner ear (cochlea). Auditory brainstem implants (ABIs) directly stimulate the hearing pathways in the brainstem, bypassing the inner ear and hearing nerve [14]. While, originally, the ABI was indicated for adults diagnosed with neurofibromatosis type 2, the device is now considered for adults and children with other nerve and inner-ear abnormalities. For current cochlear implants, individuals are able to perceive sound due to the electric current from electrodes implanted in the cochlea [14]. Whilst the sound resolution might still be improved, researchers are studying the feasibility of using light to activate the surviving neurons in the cochlea. Infrared light has been shown to offer greater sound resolution, but much work remains to optimize the technology. Further research is being conducted into utilizing the cochlear implant as a drug delivery system—when electrodes are implanted into the inner ear, insertion trauma may occur, leading to an inflammatory response [15]. Researchers continue to develop “drug-eluting” electrodes that can deliver drugs to reduce implantation and fibrotic growth and better preserve cochlear health after implantation [16]. The levels of intervention also differ anatomically, including external ear, middle ear, inner ear, and brain stem implants of today. 

We are all similar, of course, but we are also different in many aspects. The approach of personalized medicine in otology does not literally mean the creation of drugs or medical devices that are unique to a patient, but rather the ability to classify individuals into subpopulations that differ in their susceptibility to a particular disease, or in their response to a specific treatment. PM may be considered an extension of traditional approaches to understanding, treating, as well as rehabilitating a disease. Equipped with pre-, intra-, and post-operative tools that are more precise, physicians can select a therapy or treatment protocol based on a patient’s hearing or anatomical profile that may not only minimize harmful side effects and ensure a more successful outcome, but in turn also improve the patient’s quality of life as well as that of their families and peers. Medical advances are significantly increasing the amount of available disease-relevant patient data and treatment options. Some of the challenges lie in standardizing, securing, and managing this complex data. The standardization process needs to be secure and reliable since it deals with sensitive patient data. 

As hearing implant technology advances at an exponential rate, the size of both internal and external devices is expected to be miniaturized. In addition, advances in directional microphone technology will improve the design of future devices [17]. In combination, improvements in technology (including laser technology) and the utilization of robotic electrode insertion to reduce electrode insertion trauma and improve surgical accuracy [18,19,20,21,22,23,24] will achieve the goal of having a completely implantable device with a significantly improved battery life. Better speech-processing strategies will continue to improve the quality of sound perceived and improve the perception of music by cochlear implant recipients. Furthermore, bilateral implantation using a single internal device and a single processor will allow true binaural stereophonic hearing with excellent sound perception in noise [25]. Finally, continued collaborations between institutions, industry, and governments worldwide will make cochlear implantation affordable for all. Cost-saving initiatives such as more efficient and affordable microprocessors, as well as streamlining the post-operative care and rehabilitation process, may play a part in making cochlear implantation an affordable possibility for all CI candidates around the globe.

## Figures and Tables

**Figure 1:**
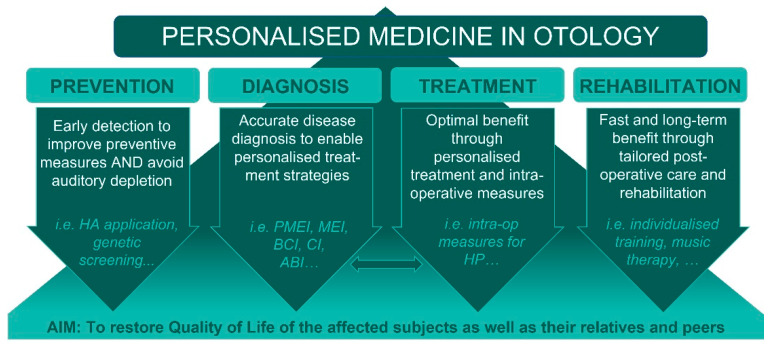
HA, Hearing Aid; PMEI, passive Middle Ear Implant; MEI, Middle Ear Implant; BCI, Bone Conduction Implant; CI, Cochlear Implant; ABI, Auditory Brainstem Implant; HP, Hearing Preservation.
